# Supplementary material for: The human metabolome and machine learning improves predictions of the post-mortem interval
Source: Nat Commun. 2026 Feb 11;17:1504. doi: 10.1038/s41467-026-69158-w (PMC12894911; doi:10.1038/s41467-026-69158-w)
Supplement: Supplementary file 1 — Supplementary Information [file 41467_2026_69158_MOESM1_ESM.pdf]

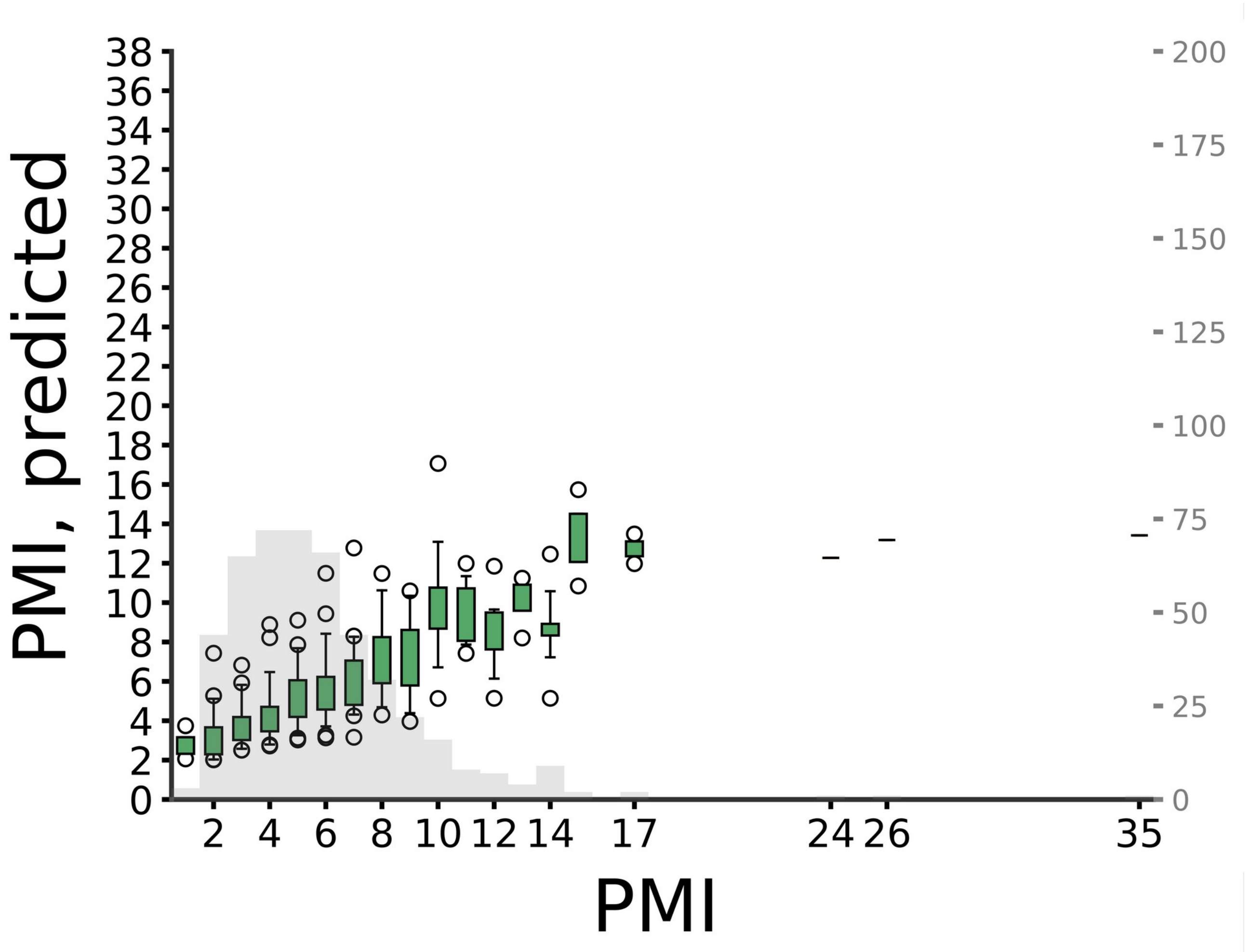

Supplementary Figure 1. *The estimations of PMI at all days present in the test data. The gray histogram shows the distribution of the data. The x-axis is the observed PMI, and the y-axis the predicted. The boxes show the median on the center line and cover the 25th–75th percentiles, with whiskers extending to the 2.5th and 97.5th percentiles.*

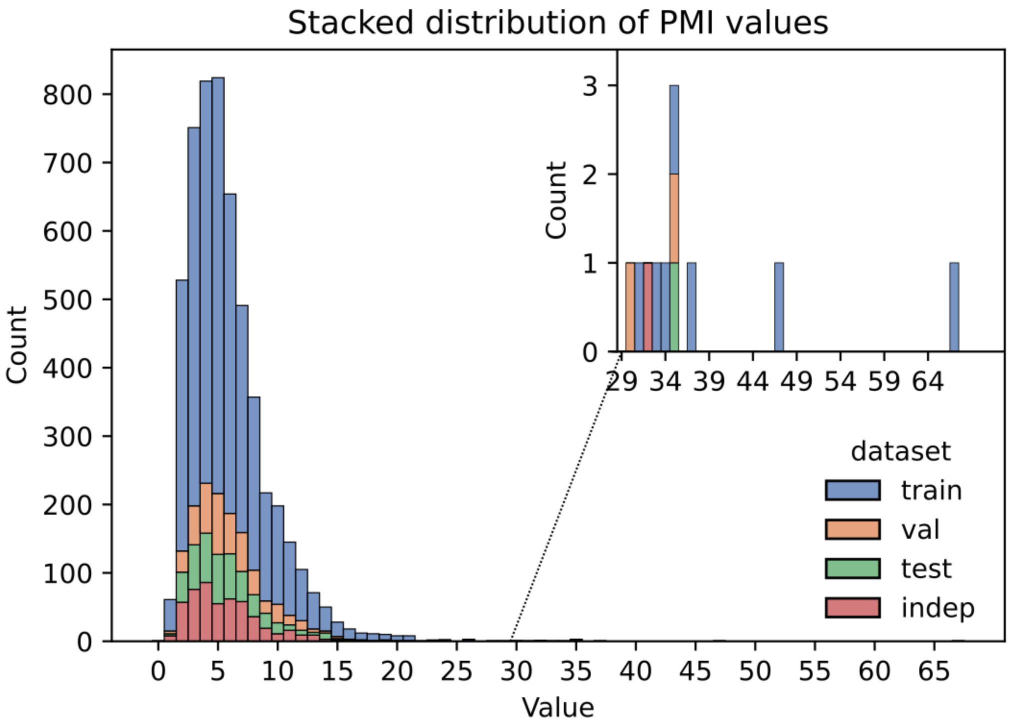

Supplementary Figure 2. *Distribution of data. Shown is the distribution of PMIs in the training, validation, test, and independent data. The histogram is stacked.*

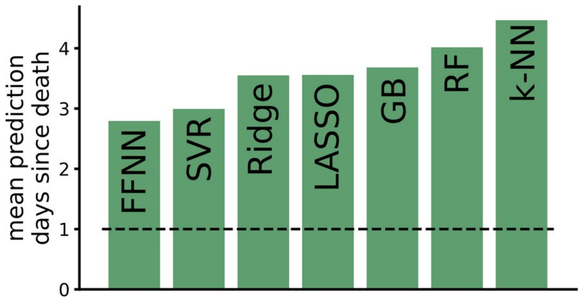

Supplementary Figure 3*. The over-estimations of the PMI at day 1, when applied to test data. The dotted line shows the target value 1, and the respective columns the mean prediction.*

Sup. Methods 1. Samples Collection and Data Acquisition

At the Swedish National Board of Forensic Medicine, approximately 6,000 autopsies are performed annually, with specimen collection taking place at several sites across the country. To ensure consistency, all procedures for collection, handling, storage, and transport are standardized. Both the forensic medicine units and the toxicology laboratory are accredited according to ISO/IEC 17020 and 17025, ensuring compliance with strict quality assurance requirements. Specimens are stored under controlled refrigerated conditions (2–8 °C) and, in general, shipped to the laboratory on the same day as the autopsy (except on Fridays), under cooled transport. Samples are typically analyzed the day after arrival. Upon arrival, they are prepared and analyzed according to standardized procedures (Roman et al., 2013). Before each analytical run, a system suitability test (SST) is performed using a panel of 10 standards. Absolute signal intensity, mass accuracy (ppm), and retention time are evaluated to confirm instrument performance. Whole blood specimens (0.25 g) are processed by protein precipitation (500 µL, ACE, EtOH, and FA) with the addition of three deuterated internal standards (amphetamine-D8, diazepam-D5, and mianserine-D3). Alongside each autopsy specimen, a blank whole blood control is prepared and injected at both the beginning and end of each analytical run. Analyses are conducted on a UHPLC-ESI-QTOF system (1290 Infinity LC, 6540 Q-TOF with JetStream interface; Agilent, Kista, Sweden). Chromatographic separation is achieved on a C18 column (Acquity HSS T3, 150 mm × 2.1 mm, 1.8 µm; Waters, Sollentuna, Sweden) with a 0.5 mL/min flow rate using gradient elution: initial 1% B for 0.6 min, followed by 5–50% B (0.7–8.0 min), 50–95% B (8–10 min, held until 11 min), and re-equilibration at 1% B (11.1–12.0 min). Mobile phase A was 10 mM ammonium formate with 0.05% FA; mobile phase B was acetonitrile with 0.05% FA. The column temperature was maintained at 60 °C. Samples admitted between July 2017 and November 2020 were run using an Agilent 6540 Q-TOF system in MS-mode only, and samples admitted afterwards usings an Agilent 6546 Q-TOF system with data-dependent acquisition (autoMS/MS). Mass spectrometric data were acquired in positive electrospray ionization mode with the following parameters: gas temperature 150 °C, gas flow 18 L/min, nebulizer 50 psi, sheath gas temperature 375 °C, and sheath gas flow 11 L/min. All specimens need to meet a SST criteria as well as internal standard requirements.

The present study is based on retrospectively collected whole blood samples analyzed as part of routine forensic toxicological screening over multiple years. In total, n = 5388 autopsy cases were included, each corresponding to a unique biological sample analyzed once. No technical replicates or pooled quality control samples were available due to the retrospective nature of the dataset.

Supplementary References

Roman, M., Ström, L., Tell, H., & Josefsson, M. (2013). Liquid chromatography/time-of-flight mass spectrometry analysis of postmortem blood samples for targeted toxicological screening. *Analytical and bioanalytical chemistry*, *405*(12), 4107–4125. https://doi.org/10.1007/s00216-013-6798-0
