# Supplementary material for: The human metabolome and machine learning improves predictions of the post-mortem interval
Source: Nat Commun. 2026 Feb 11;17:1504. doi: 10.1038/s41467-026-69158-w (PMC12894911; doi:10.1038/s41467-026-69158-w)
Supplement: Supplementary file 2 — Description of Additional Supplementary Files [file 41467_2026_69158_MOESM2_ESM.pdf]

The Description of Additional Supplementary Files

Supplementary Data 1: Excel sheet containing tested values of the hyperparameter optimization, and the corresponding performance.

Supplementary Data 2: Excel sheet of named metabolomic features for which the intensity decreased with PMI-pseudo-time and that were used by the model.

Supplementary Data 3: Excel sheet of named metabolomic features for which the intensity increased with PMI pseudo-time and that were used by the model.

Supplementary Data 4: Excel sheet of named metabolomic features for which the intensity showed a complex pattern over PMI pseudo-time and that were used by the model.

Supplementary Data 5: The used settings of the alternative machine learning methods
